# Supplementary material for: Interface confined hydrogen evolution reaction in zero valent metal nanoparticles-intercalated molybdenum disulfide
Source: Nat Commun. 2017 Feb 23;8:14548. doi: 10.1038/ncomms14548 (PMC5331331; doi:10.1038/ncomms14548)
Supplement: Supplementary Information — Supplementary Figures, Supplementary Tables, Supplementary Notes, Supplementary Methods and Supplementary References [file ncomms14548-s1.pdf]

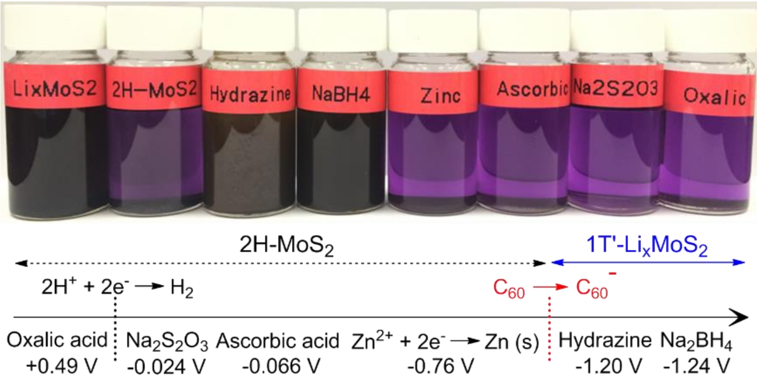

Supplementary Figure 1. Chemical reduction of C_{60} with 1T'-Li_xMoS_2, 2H-MoS_2 and other common reducing agents. Li_xMoS_2, hydrazine and sodium borohydride are able to reduce C_{60} to C_{60}^- at r.t. The black coloration is evident of reduction process where C_{60}^- (fulleride) ions form.

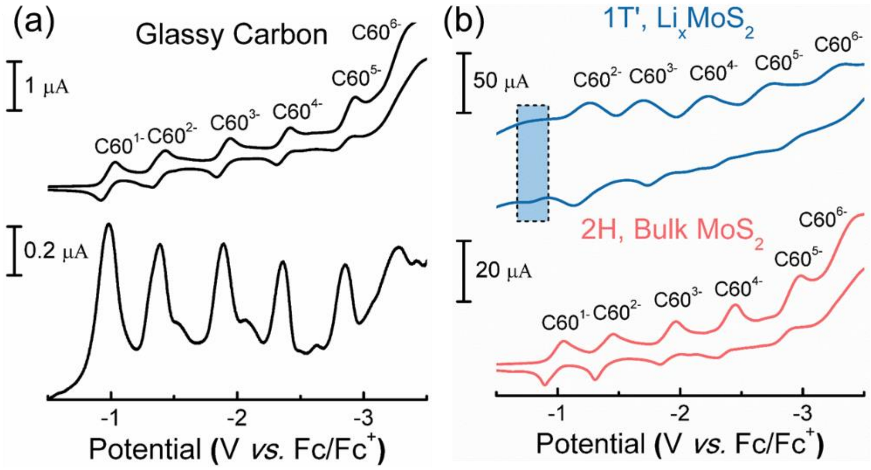

Supplementary Figure 2. Electrochemical reduction of C_{60}: (*A*) DPV and CV curves on glassy carbon (GC), (*B*) CV curves on bulk, 2H-MoS_2 and 1T'-Li_xMoS_2 in 1:5.4 AN/Toluene at -15°C.

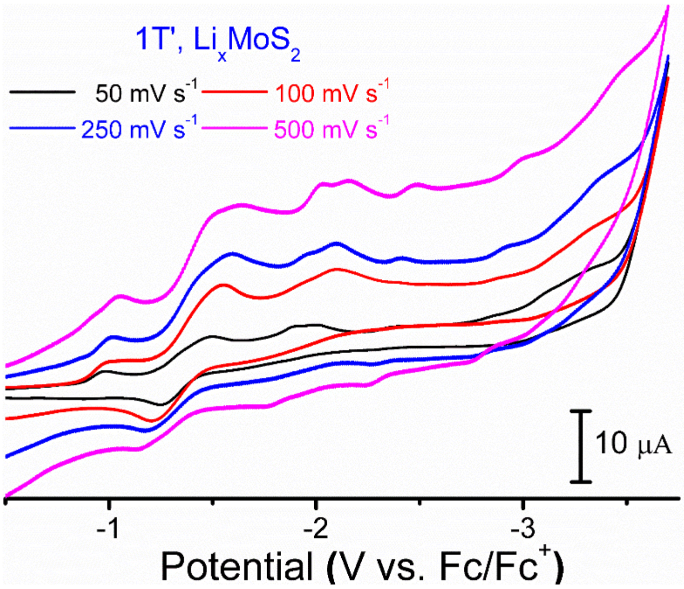

Supplementary Figure 3. Electrochemical reduction of C_{60} on 1T'-Li_xMoS_2 at various scanning rates: CVs at 50, 100, 250 and 500 mV/s;

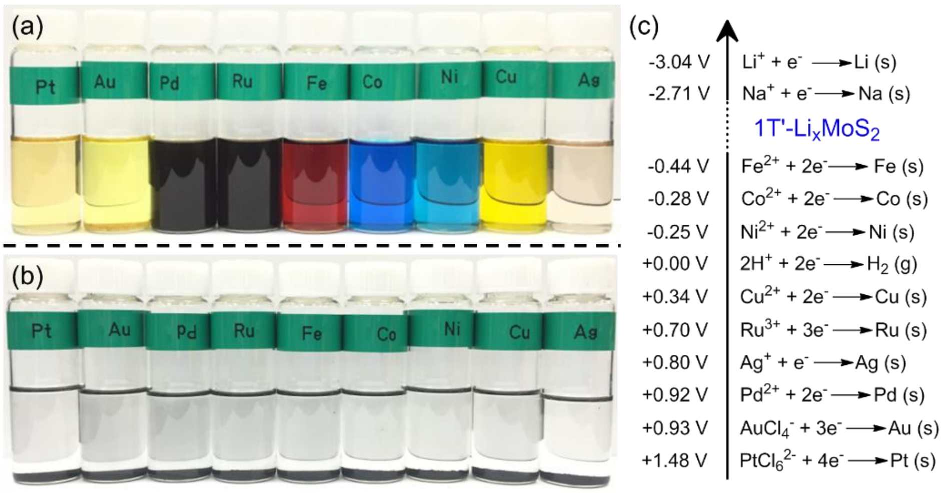

Supplementary Figure 4. Metal ion precursor solutions (*A*) before and (*B*) after reduction with 50 mg Li_xMoS_2 at 80 ^{o}C for 2 days. (*C*) Standard electrode potential of these metal ions. From left to right: Na_2PtCl_6•$6H_2O$ (17.6 mg in THF), $HAuCl_4$•$4H_2O$ (15.8 mg in THF), $PdCl_2$ (27.7 mg in NMP), $RuCl_3$•xH_2O (17.6 mg in NMP), $Fe(OAc)_2$ (27.2 mg in NMP), $CoCl_2$ (20.3 mg in NMP), $NiCl_2$ (20.3 mg in NMP), $CuCl_2$ (21.0 mg in NMP) and $AgNO_3$ (53.1 mg in NMP). It is seen that all metal ion precursors have been reduced to their zero-valent state.

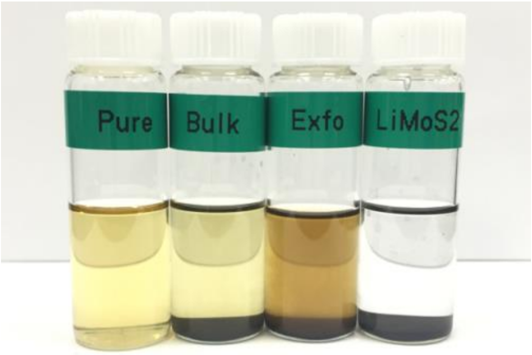

Supplementary Figure 5. Comparison in reducing power of 2H and 1T' phase with Na_2PtCl_6. From left to right: pristine Na_2PtCl_6•$6H_2O$ solution (17.6 mg in THF), with 50 mg bulk MoS_2, exfoliated MoS_2 and Li_xMoS_2 after heating at 80 ^oC for 2 days. Exfoliated MoS_2 nanosheets only show weak reducing power due to the loss of 1T'-phase after water exfoliation.

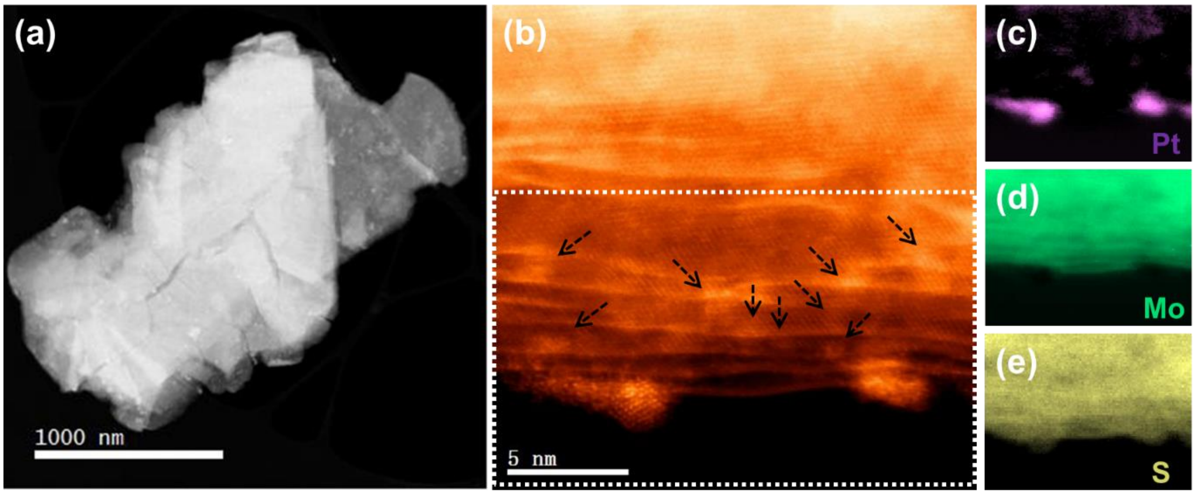

Supplementary Figure 6. (*A*) Dark field TEM image of Pt-MoS_2. (*B*) High resolution HAADF-STEM image and (*C-E*) EELS mapping showing the intercalation of Pt nanoparticles with an average size of ~ 2 nm in between MoS_2 layers (as indicated by black arrows). Since this is a cross-section image, we are only able to image those nanoparticles at the edges, whereas intercalated nanoparticles (especially for those located on the central plane) in the inner regions cannot be imaged due to the low TEM contrast.

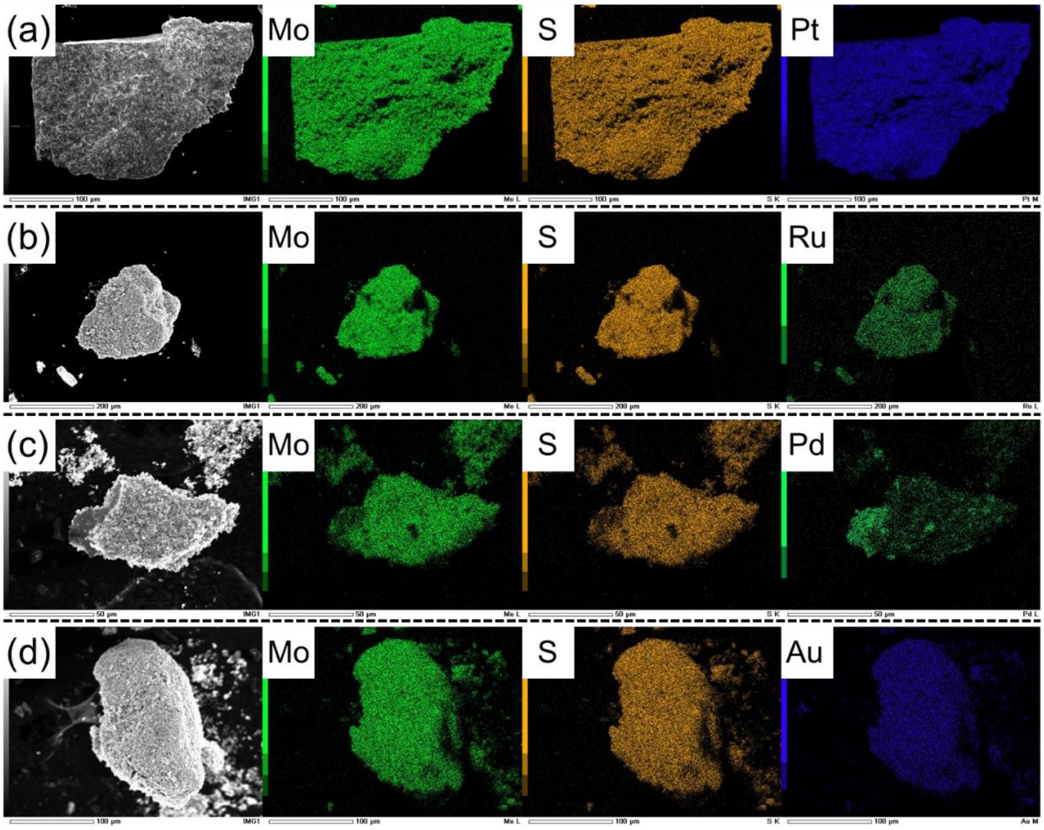

Supplementary Figure 7. Uniform distribution of intercalated metals on MoS_2. FESEM EDS mapping of (*A*) Pt-MoS_2, (*B*) Ru-MoS_2, (*C*) Pd-MoS_2 and (*D*) Au-MoS_2. Scale bar: (*A*) 100 μm, (*B*) 200 μm, (*C*) 50 μm and (*D*) 100 μm.

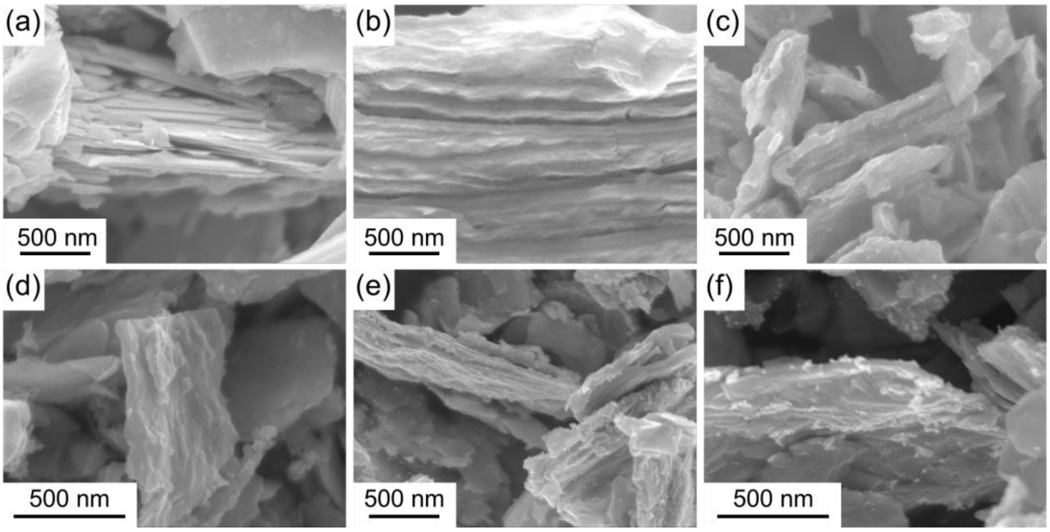

Supplementary Figure 8. Expanded morphology after n-BuLi and metal intercalation. FESEM images of (*A*) bulk MoS_2, (*B*) Li_xMoS_2, (*C*) Pt-MoS_2, (*D*) Ru-MoS_2, (*E*) Pd-MoS_2 and (*F*) Au-MoS_2. The ordered layered structure of MoS_2 was slightly distorted after intercalation of noble metals.

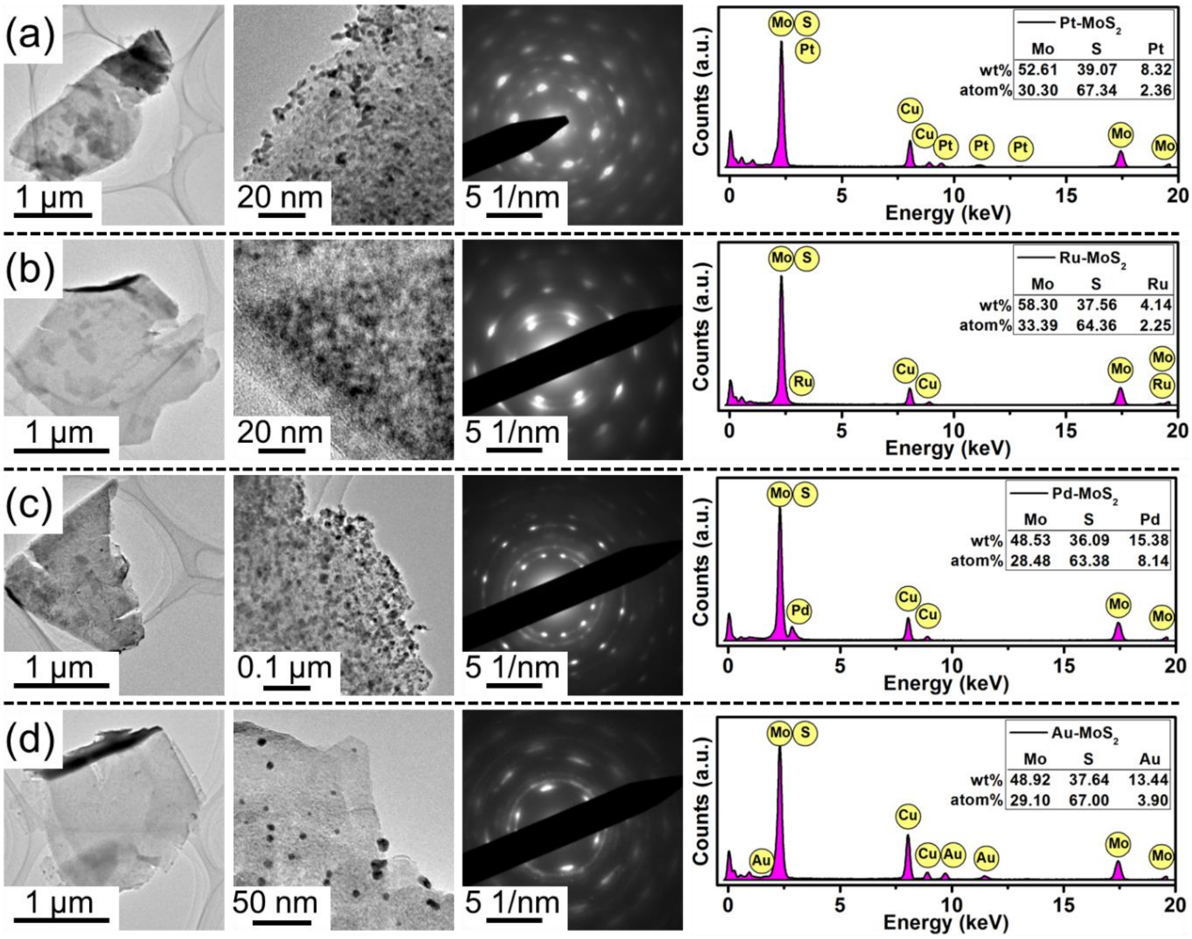

Supplementary Figure 9. TEM characterization of metal intercalated MoS_2: TEM images, SAED patterns and EDS spectra of (*A*) Pt-MoS_2, (*B*) Ru-MoS_2, (*C*) Pd-MoS_2 and (*D*) Au-MoS_2. The SAED pattern of Li_xMoS_2 (not shown here) also confirm the existence of 1T'-phase by the presence of the ~5.6 Å, 2 × 1 superstructure spots.

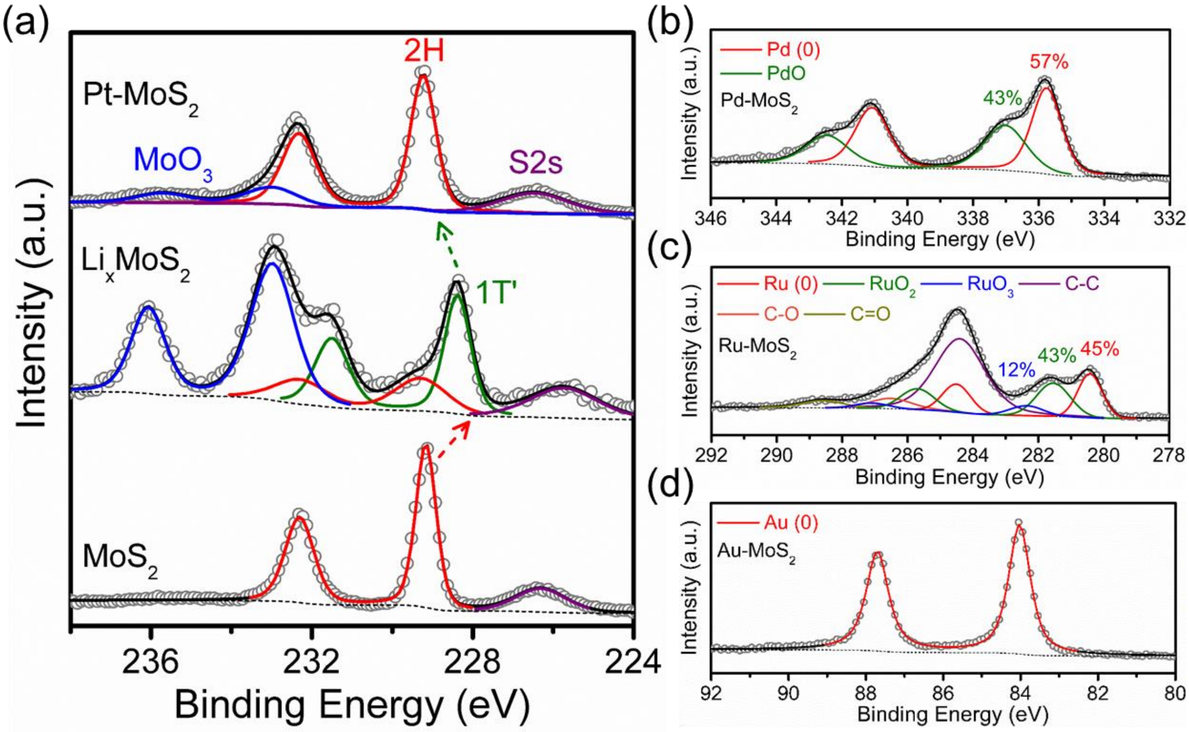

Supplementary Figure 10. XPS characterization of metal intercalated MoS_2. (*A*) Phase evolution from Mo_{3d} spectra of 2H, bulk MoS_2, 1T'-Li_xMoS_2 and 2H, Pt-MoS_2; (*B*) Pd_{3d}, (*C*) Ru_{3d} and (*D*) Au_{4f} spectra showing the zero valent state of corresponding intercalated metals. We found some oxidized Pd species, which may be ascribed to their different chemical environments (sit on surface, edge or intercalated).

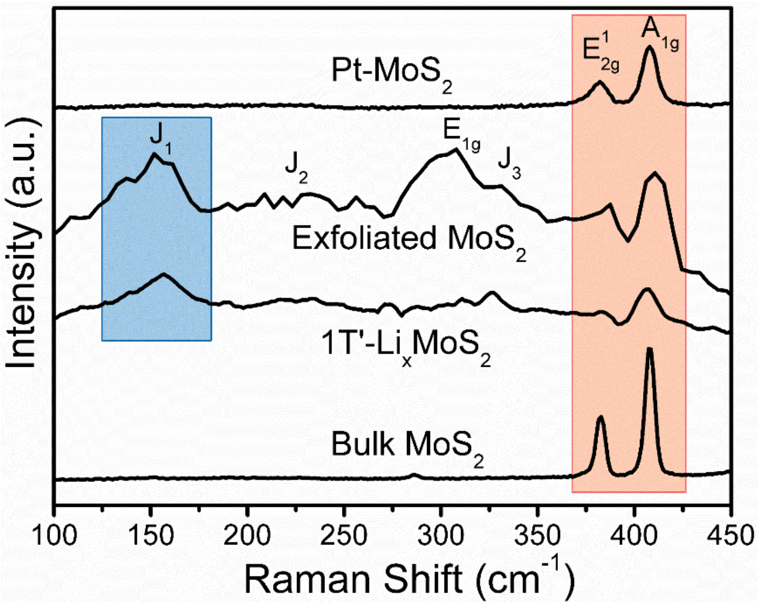

Supplementary Figure 11. Raman spectra of bulk, 2H-MoS_2, 1T′-Li_xMoS_2, 1T′-exfoliated MoS_2 and 2H, Pt-MoS_2, showing evidence of the phase conversion.

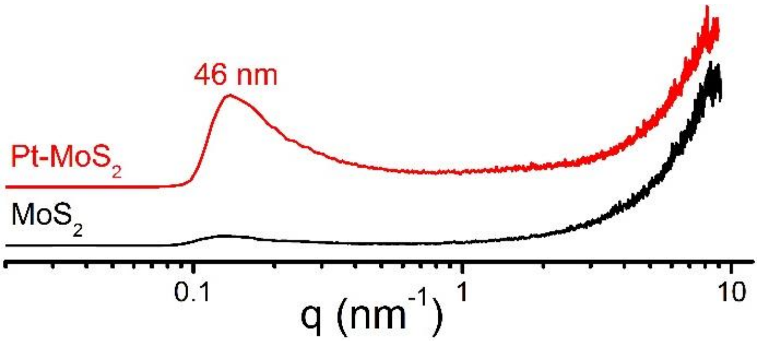

Supplementary Figure 12. Small-angle X-ray scattering (SAXS) profiles of MoS_2 and Pt-MoS_2. The products of the scattering intensity, I, and the inverse of form factor of a flat, thin sheet (q^2), $I*q^2$, were plotted against the scattering vector modulus, q.

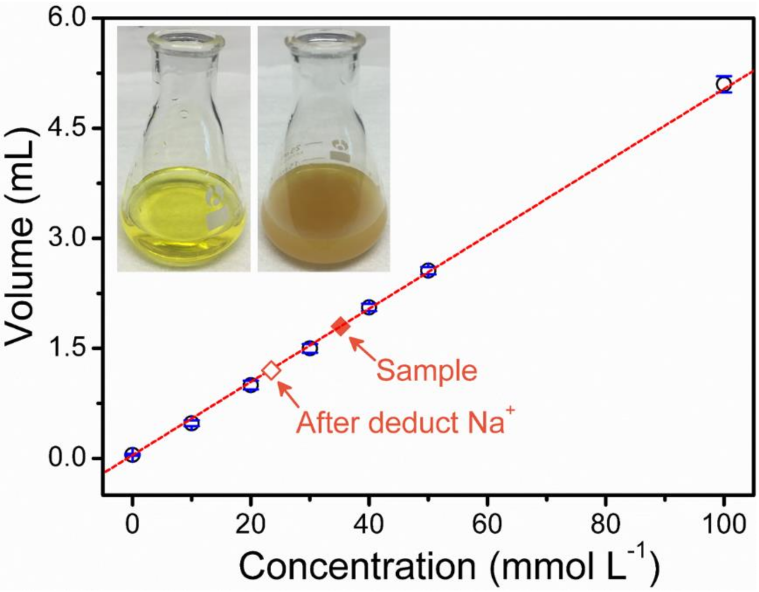

Supplementary Figure 13. Mohr titration of chloride in the supernatant of Pt-MoS_2.

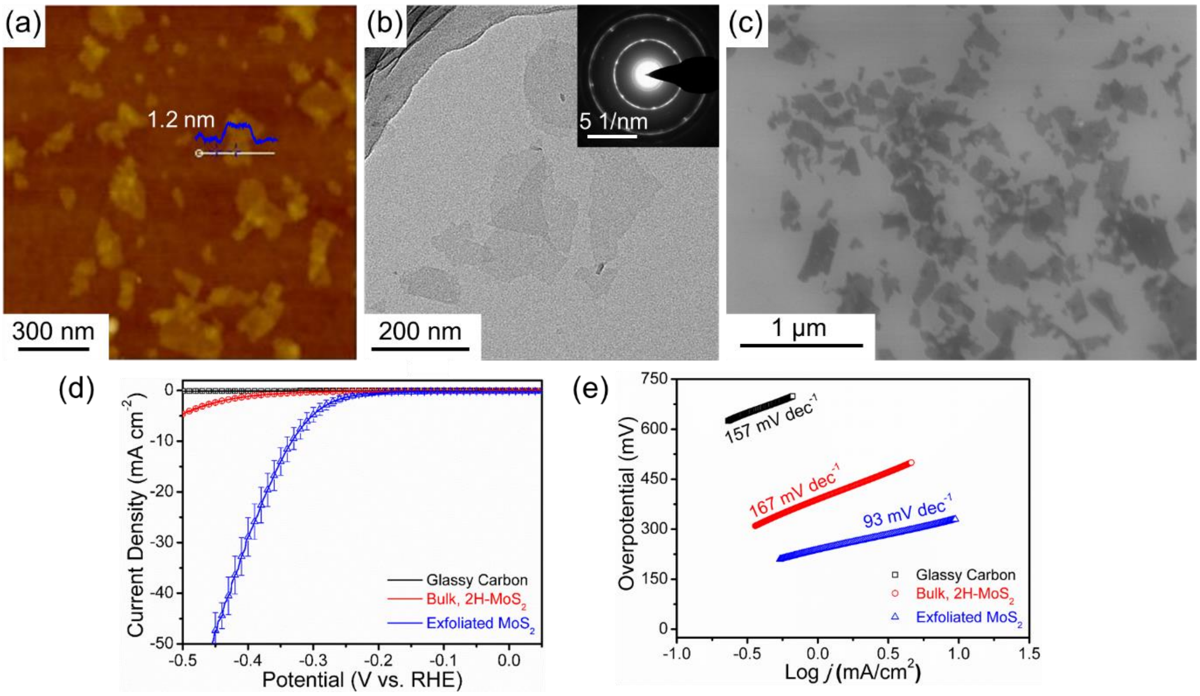

Supplementary Figure 14. Monolayer MoS_2 nanosheets from n-BuLi exfoliation: (*A*) AFM, (*B*) TEM and (*C*) FESEM images of exfoliated MoS_2 (inset: SAED pattern). HER Performance of exfoliated MoS_2: (*D*) LSV curves and (*E*) corresponding Tafel slopes.

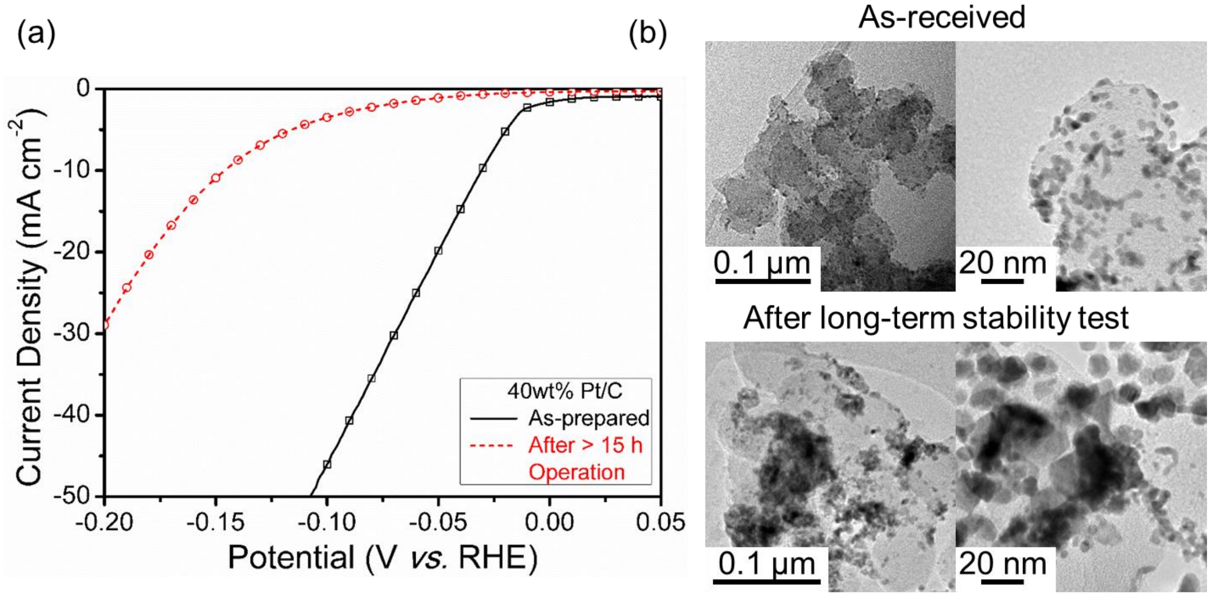

Supplementary Figure 15. Characterization of commercial 40 wt% Pt/C catalyst. (*A*) LSV curves before and after > 15 hours continuous operation at 50 mA cm^{-2}. (*B*) TEM images of as-received and tested Pt/C catalysts.

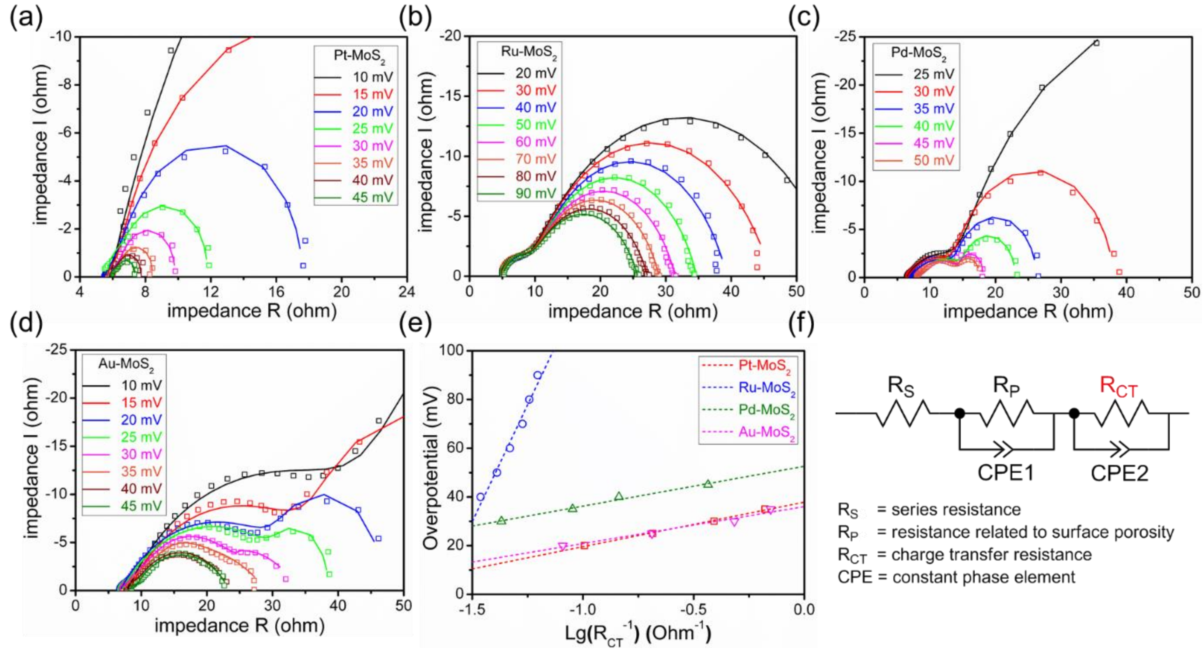

Supplementary Figure 16. EIS spectra of metal intercalated MoS_2 at various overpotentials. (*A*-*D*) Nyquist plots of Pt, Ru, Pd, Au-MoS_2, (*E*) semi-logarithmic plot of applied overpotential *vs.* R_{ct}, and (*F*) simulation model.

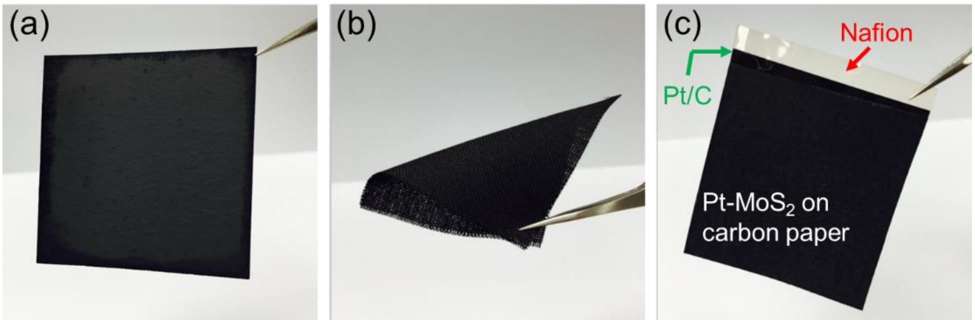

Supplementary Figure 17. Digital photos of a 25 cm^2 membrane-electrode assemblies using Pt-MoS_2 catalysts: (*A*) coating onto carbon paper, (*B*) coating onto carbon cloth, (*C*) combined with Nafion® membrane and commercial 40% Pt/C gas diffusion electrode, all at a loading of 1 mg/cm^2.

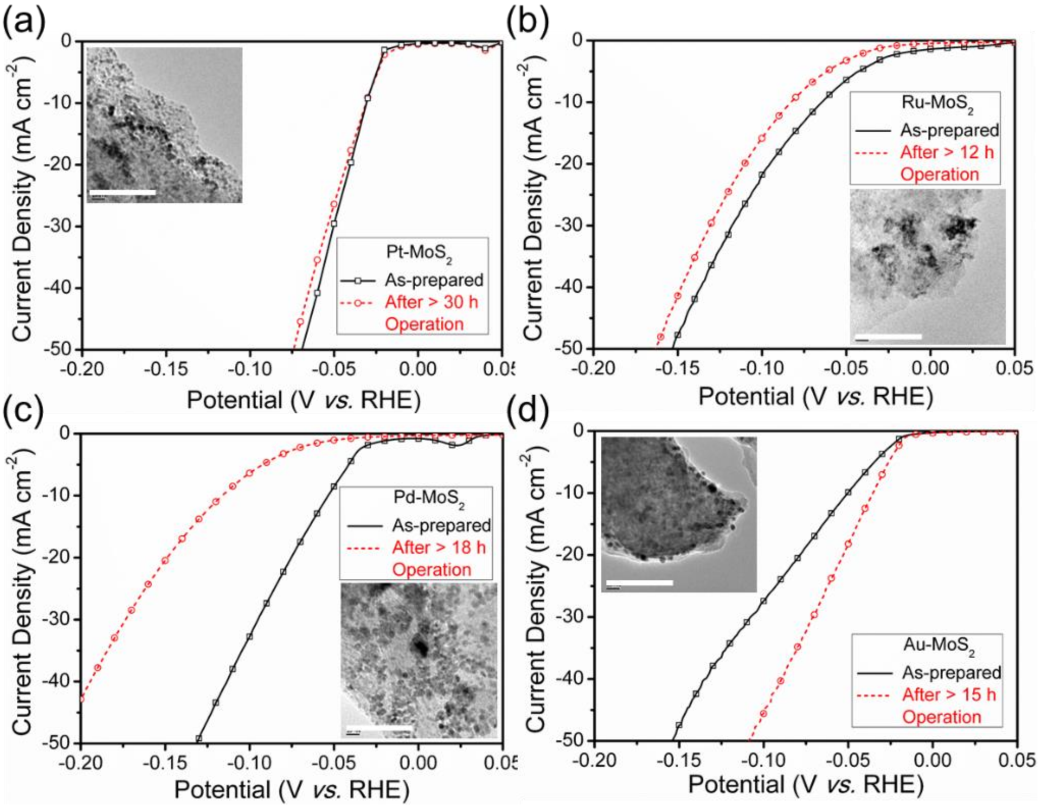

Supplementary Figure 18. HER Stability after > 15 or 30 hours continuous operation at 50 mV cm^{-2}. (*A*-*D*) LSV curves of Pt, Ru, Pd, Au-MoS_2 before and after long-term operation in 0.5 M H_2SO_4. Inset: corresponding TEM image after test. Scale bar: (*A*) 50 nm, (*B*) 50 nm, (*C*) 50 nm and (*D*) 100 nm, respectively.

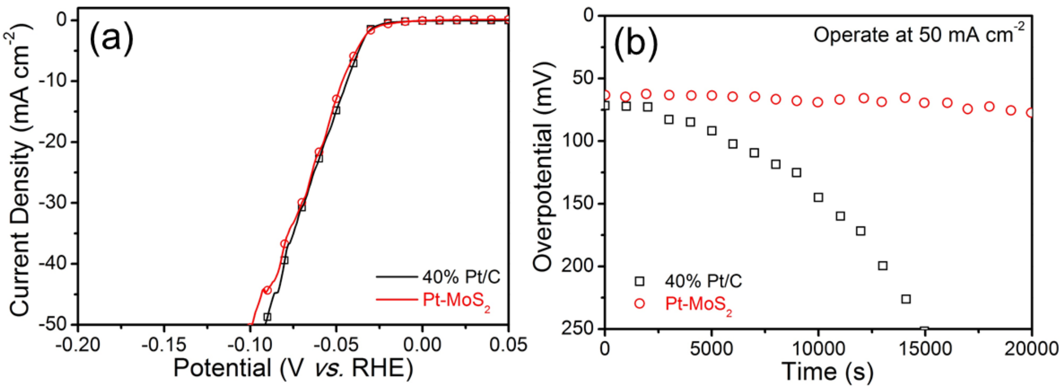

Supplementary Figure 19. HER measurements with a 3-electrode H-cell using a saturated Hg/$HgSO_4$ electrode, a carbon rod and a Nafion-117 membrane as the reference, the counter electrode and the separated membrane. (*A*) LSV Curves (*B*) Stability test. There's no major difference in HER activity and stability using carbon counter and platinum counter.

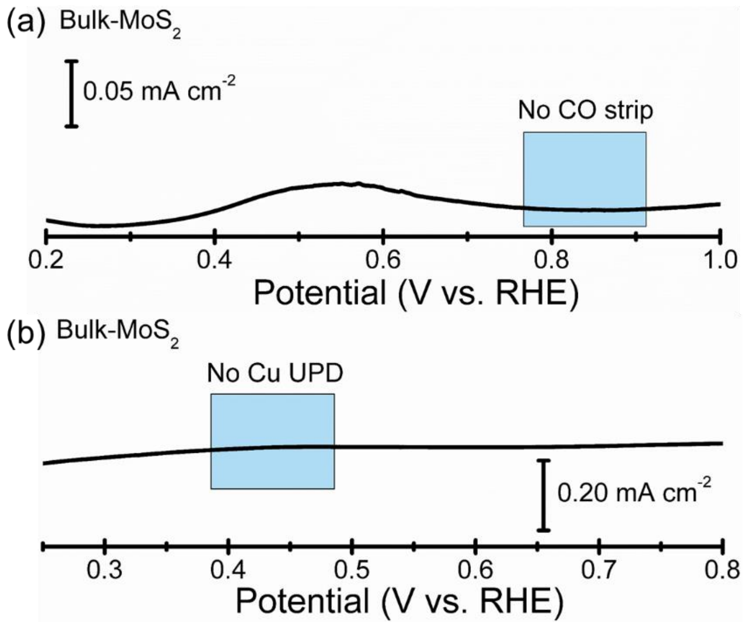

Supplementary Figure 20. Bulk, 2H-phase MoS_2 is insensitive to both (*A*) CO stripping and (*B*) Cu UPD.

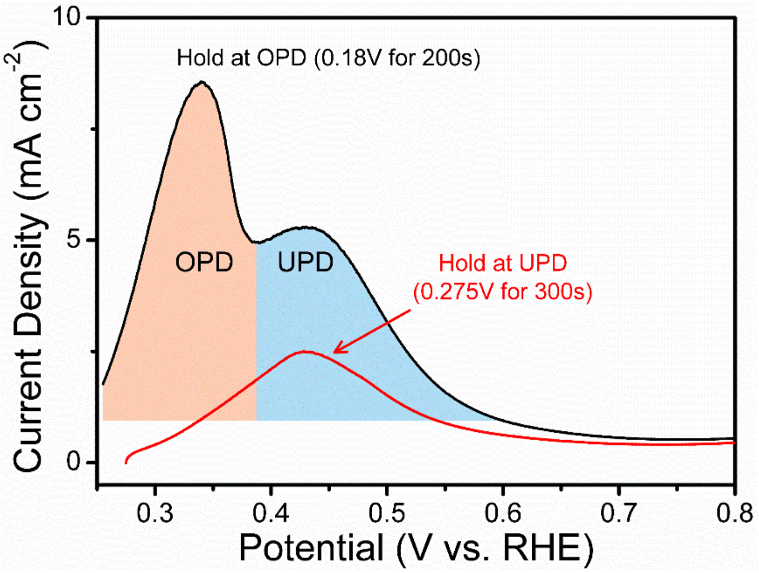

Supplementary Figure 21. Representative Cu UPD and OPD peaks on Pt-MoS_2, from which we can rule out the influence of Cu OPD on our experiments.

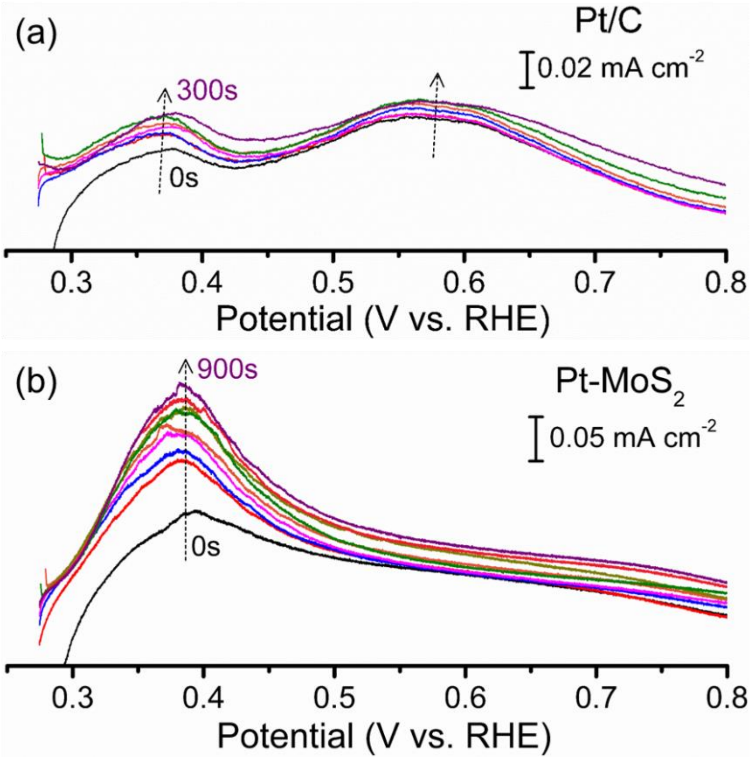

Supplementary Figure 22. Cu UPD with different holding times at 275 mV *vs.* RHE of (*A*) 40 wt% Pt/C and (*B*) Pt-MoS_2. Pt/C shows the saturation of monolayer deposition (> 90%) after 30 s while the saturation for Pt-MoS_2 can be as long as 15 mins.

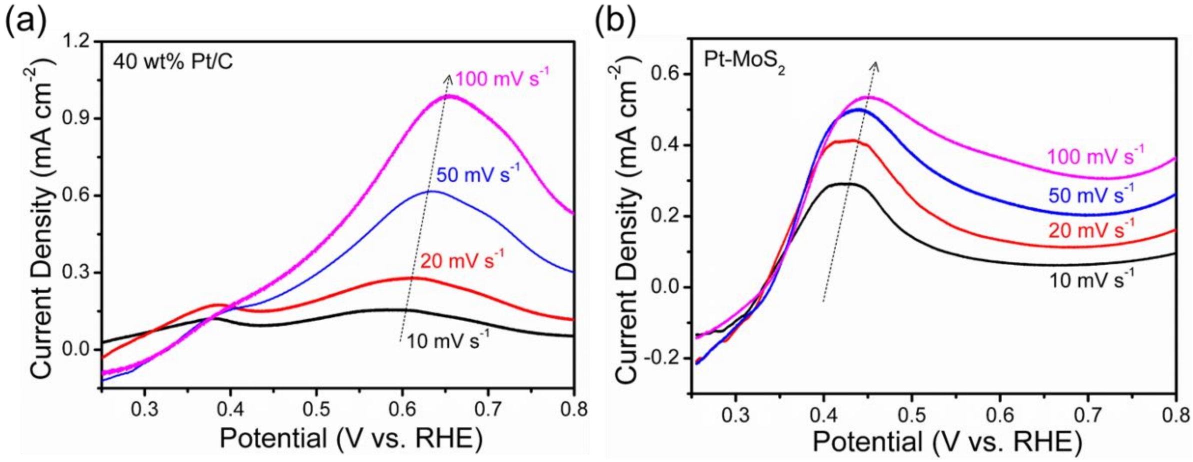

Supplementary Figure 23. Cu UPD at different scan rates of (*A*) 40 wt% Pt/C and (*B*) Pt-MoS_2 without holding prior to test. We also conducted the same experiments with 300 s holding (Supplementary Figure 21). In that case, the ECSA values of Pt-MoS_2 at different scan rates are similar due to monolayer saturation.

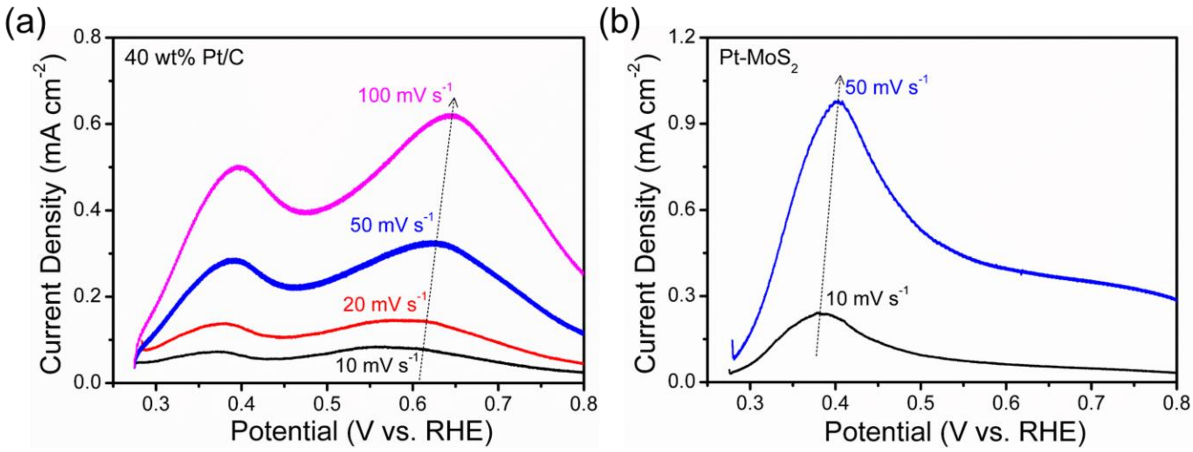

Supplementary Figure 24. Cu UPD at different scan rates of (*A*) 40 wt% Pt/C and (*B*) Pt-MoS_2 after monolayer saturation (300 s holding time at 275 mV *vs.* RHE).

Supplementary Table 1. Half-Wave Potential ($E_{1/2}$) of C_{60}. [1]

Electrode	E_1	E_2	E_3	E_4	E_5	E_6
GC	-0.98	-1.38	-1.89	-2.37	-2.86	-3.26
2H-MoS_2	-0.97	-1.38	-1.90	-2.37	-2.88	-3.31
1T'-Li_xMoS_2	-0.80 [2]	-1.20	-1.71	-2.24	-2.78	-3.27

[1.] All potential are referenced to the Fc/Fc^+ couple. $E_{1/2}$ is the average value of the oxidation and reduction peaks potential.

[2.] This redox peak is very small in the CV/DPV.

Supplementary Table 2. Elemental composition of MoS_2 samples.

Sample	Elemental Analysis (EA) / wt% [1]					EDS / wt%	
	Moly	Sulfur	Intercalated Metal	Li^+ / Cl^- [2]	Theoretical Value	SEM Map	TEM
MoS_2	60.34	39.66	--	--	--	--	--
Li_xMoS_2	49.44	26.83	4.11 / Li	--	4.16 / Li	--	--
Exfoliated MoS_2	43.14	26.00	--	< 0.5	--	--	--
Pt-MoS_2	40.82	26.44	10.43 / Pt	< 0.5	10.90 / Pt	9.38 / Pt	8.32 / Pt
Ru-MoS_2	45.30	26.75	8.65 / Ru	< 0.5	9.50 / Ru	9.12 / Ru	4.14 / Ru
Pd-MoS_2	32.95	21.18	24.46 / Pd	< 0.5	24.95 /Pd	21.02/Pd	15.38 /Pd
Au-MoS_2	41.60	23.48	18.26 / Au	< 0.5	17.00/ Au	19.39/Au	13.44 /Au

Sample	XPS / atom%				XPS
	Moly	Sulfur	Intercalated Metal	S/Mo Ratio	Mo_{3d} Peak Position / eV
MoS_2	34.7	65.3	--	1.88	229.2, 232.3, 226.3
Li_xMoS_2	32.5	67.5	Not accurate [3]	2.08	227.7, 230.9 (1T′), 229.1, 232.2 (2H), 232.3, 235.4, 225.0
Exfoliated MoS_2	34.3	65.7	--	1.92	228.5, 231.7 (1T′), 229.2, 232.4 (2H), 225.8
Pt-MoS_2	31.6	64.3	4.1 / Pt	2.03	229.2, 232.3, 233.0, 235.7, 226.5
Ru-MoS_2	29.6	56.4	14.0 / Ru [3]	1.91	229.1, 232.2, 232.8, 235.5, 226.3
Pd-MoS_2	30.4	58.7	10.9 / Pd	1.93	229.1, 232.3, 232.8, 235.6, 226.3
Au-MoS_2	34.4	61.0	4.6 / Au	1.77	229.1, 232.2, 232.8, 235.6, 226.2

1. Mo, Li, Pt, Ru, Pd and Au were determined by ICP-OES; S was determined by both CHNS analysis and anionic ion-exchange chromatography (IC) of SO_4^{2-}.

2. Residual ions were completely leached out in our catalysts as proven by ICP-OES and IC.

3. Only Mo, S and corresponding metal were analyzed by EDS and XPS. The composition of Li_xMoS_2 and Ru-MoS_2 were not accurate due to the low sensitivity to Li_{1s} and the overlap between Ru_{3d} and C_{1s} in XPS. 1T′ peaks shifted to higher positions in exfoliated MoS_2 due to the removal of counter ions (Li^+).

Supplementary Table 3. ECSAs from CO Stripping and Cu UPD.

Sample	Method	Scan Rate (mV s^{-1})	Holding Time (s)	ECSA (cm^{-2})
40 wt% Pt/C	CO Strip	10	600	0.620
Pt-MoS_2				0.160
40 wt% Pt/C	Cu UPD	10	0	0.445
			8	0.487
			15	0.500
			30	0.518
			60	0.532
			120	0.545
			300	0.584
Pt-MoS_2	Cu UPD	10	0	0.461
			15	0.662
			30	0.710
			60	0.762
			120	0.827
			300	0.853
			600	0.970
			900	1.021
		50	300	0.851

Supplementary Table 4. Summary of recently reported HER catalysts

Catalyst	Loading (mg cm^{-2})	Onset-potential (mV)	Overpotential at 10 mA cm^{-2} (mV)	Tafel (mV $decade^{-1}$)	Stability (cycle or hour)	Ref.
MoS_2 NSs	0.05	~100	~200	40	> 150 cycles	1
MoS_2 NSs	Unknown	~54	~200	~100	~ 3,000 cycles	2
MoS_2 NSs	Unknown	~150	187	43	~ 1,000 cycles	3
MoS_2 NPs	0.3	~95	190	60-65	~ 1,000 cycles	4
Vertically Aligned MoS_2 Film	0.022	~ 175	216	43-47	~ 1,000 cycles	5
$[Mo_3S_{13}]^{2-}$/ GP	0.1	100	180	38-40	~ 1,000 cycles	6
Double-gyroid MoS_2	Unknown	150	240	50	Unknown	7
Mo_2C/CNTs	2.0	~0	152	55	~ 3,000 cycles	8
CoN_x/C	2.0	20	133	57	~ 5,000 cycles	9
UHV MoS_2/ Au (111)	Mono-layer	~100	Unknown	55-60	Unknown	10
MoC_x	Unknown	~ 25	142	53	Unknown	11
Pt-Pd-rGO	0.05 for metals	~20	~25	~22	~5,000 cycles	12
1 wt% Au-MoS_2	Unknown	~90	~255	71	Unknown	13
Pt-TiS_2	0.1	~25	~75	41	~1,000 cycles	14
36 wt% Pt-MoS_2	0.075	~20	~50	~40	Unknown	15
1.7 wt% Pt-MoS_2	1.0	~50	~145	96	~ 5,000 cycles	16
**10 wt% Pt-MoS_2**	**0.07**	**~22**	**~35**	**~25**	**> 30 hours**	**This Work**

Supplementary Note 1

The corresponding d-spacing values were calculated using the formula $d = 2\pi/q$. Pt-MoS_2 was prepared using single-crystal MoS_2. *(17)* The SAXS profiles confirm a loosely stacked structure of Pt-MoS_2 due to the hydrogen-bubbles induced volume expansion during zero-valent intercalation.

Supplementary Note 2

Silver nitrate standard solution (0.100 M) was prepared and calibrated by NaCl standard solution. Samples were adjusted to pH 7-9 and mixed with 1 mL of 5% K_2CrO_4 solution (indicator). After all the chloride has been precipitated as white silver chloride, the formation of a silver chromate precipitate (brown-red), as shown in the inset photo ($LiCl + AgNO_3 = AgCl\downarrow + LiNO_3$; $2AgNO_3 + K_2CrO_4 = 2KNO_3 + Ag_2CrO_4\downarrow$). Every sample was measured for 3-5 times to calculate the average values and standard deviations. We measured a concentration of 35.2 mmol L^{-1} of Cl^- (23.5 mmol L^{-1} after deducting Na^+) in the supernatant of Pt-MoS_2 after reaction. This corresponds to > 80% of total Li^+ ions in Li_xMoS_2 if we consider Li^+ as the only counter ions of Cl^- (exclude Na^+ in the case of Na_2PtCl_6). Note that $PtCl_6^{2-}$ has been completely reacted with Li_xMoS_2 (Supplementary Figure 4, clear supernatant), if Li^+ ions are not exchanged outside, Cl^- ions should be remained in Pt-MoS_2 powders to compensate the excess charge. In this case, we believe that Li^+ ions are mostly ion-exchanged from MoS_2 host after zero-valent intercalation.

Supplementary Note 3

There are four major degradation mechanisms for Pt catalysts. *I.* Pt dissolution and redeposition, such as $Pt \rightarrow Pt^{2+} + 2e^-$ (platinum dissolution), $Pt + H_2O \rightarrow PtO + 2H^+ +2e^-$ (platinum oxide film formation), $PtO + 2H^+ \rightarrow Pt^{2+} + H_2O$ (chemical dissolution of platinum oxide); *II.* Coalescence of Pt nanoparticles by Pt nano-crystallite migration on the support surface; *III.* Pt nanoparticle agglomeration caused by corrosion of carbon support, *e.g.*, $R\text{-}C_s\text{-}H \rightarrow R\text{-}C_s\text{-}OH \rightarrow R\text{-}C_s\text{=}O \rightarrow R\text{-}C_sOOH \rightarrow R\text{-}H + CO_2$ (g); and *IV.* Catalyst deactivation of contaminant intermediates. As a result, the diameter of Pt nanoparticles after long-term stability test are much larger than that of as-received Pt/C. This is the major reason for the degraded performance of commercial Pt/C catalysts. *(18, 19)*

Supplementary Note 4

The strong dependence of R_{CT} on the overpotential in both Tafel and semi-logarithmic plots suggests the Volmer-Tafel process of hydrogen evolution in our samples. *(20)*

Supplementary Note 5

Noble metals intercalated-MoS_2 are quite stable towards continuous operation while commercial Pt/C is rapidly degraded. Such improved stability can be attributed to 1) the inherently excellent mechanical resistance and stability of bulk, 2H-MoS_2 in an acidic and electroactive environment, thus it is very unlikely to be damaged by the H_2 bubbles produced from the intercalated nanoparticles; 2) the anchoring effect by adjacent MoS_2 layers that

significantly reduces the dissolution or migration of Pt nanoparticles during operation, which is similar to the carbon coating methods in the literature *(18, 19)*. In such case, the leaching of Pt nanoparticles is not favorable as demonstrated by the stable HER performance and the TEM images in Supplementary Figure 16. The relatively poor stability of Pd-MoS_2 can be ascribed to the partial oxidation of Pd nanoparticles as evidenced by XPS spectra in Supplementary Figure 9*B (21)* since PdO has a much higher solubility in acidic condition.

Supplementary Note 6

Cu UPD is unique for its full, monolayer coverage on noble metal surface while Cu OPD (over-potential deposition, also known for bulk deposition) can happen on any surface including bulk MoS_2 with multilayer deposition. Therefore, one should carefully examine the contribution from Cu OPD when using Cu UPD to calculate the ECSA of Pt-based catalysts.

Based on the Nernst equation, equilibrium potential for Cu bulk deposition in 0.5 M H_2SO_4 with 1 mM Cu^{2+} is *ca.* 0.25 V *vs.* RHE. From a thermodynamic point of view, UPD and OPD of Cu will take place on the potential region more positive and negative than 0.25 V. As shown in Supplementary Figure 19, if we hold at a OPD potential (*e.g.,* 0.18 V *vs.* RHE), we can observe the Cu OPD peak at around 0.34 V *vs.* RHE as well as the Cu UPD peak at 0.42 V. However, this OPD peak is not observed when we hold at a UPD potential (*e.g.,* 0.275 mV *vs.* RHE), suggesting only Cu UPD happens in such condition. *(19)* Because Cu UPD is unique to noble metals, the peak at 0.42 V should not be observed in bulk MoS_2 as demonstrated in Supplementary Figure 17*B*. In contrast, we can observe the OPD peak at 0.34 V for bulk MoS_2 (not shown here).

To further confirm this Cu UPD peak, we performed Cu UPD with various holding time in Figure 5*C* and Supplementary Figure 20. The Cu UPD will finally reach a saturation because of its full, monolayer coverage on active Pt sites. In contrast to Cu UPD, we didn't observe any saturation of OPD peak even after 300 s. The calculated ECSA from Cu OPD (3.94 cm^2) is also much higher than that from Cu UPD (0.85 cm^2) or from the literature. *(22, 23)*

Finally, this Cu UPD peak is supported by its scan rate dependence in Supplementary Figure 22 and Supplementary Table 3. The ECSAs at various scan rates after monolayer saturation should be similar (*e.g.,* 0.851 *vs.* 0.852 cm^2), while Cu OPD never get similar results due to the nature of multilayer deposition.

Based on all these observations, we conclude the peak at ~ 0.42 V *vs.* RHE in Pt-MoS_2 should be Cu UPD peak and the mismatched ECSAs from CO stripping and Cu UPD may attribute to the different accessibility of inner and outer Pt nanoparticles to CO gas and cupric ions.

Supplementary Methods

20 mL n-BuLi (1.6 M in hexane, 32 mmol) in hexane was added to 1 g (6.25 mmol) dry MoS_2 powder (< 2 μm) in an Argon filled glove box. The dispersion was stirred at room temperature for 2 days. The black product (Li_xMoS_2) obtained was washed repeatedly with anhydrous hexane at 3,000 rpm for 5 mins to remove unreacted n-BuLi and other soluble impurities. 33.7 mg (0.06 mmol) $Na_2PtCl_6·6H_2O$ (98%) was added to 100 mg Li_xMoS_2 (~0.6 mmol) with 20 mL anhydrous THF in glove box. After the decrease of hydrogen gas formation, the mixture was sealed in a Teflon®-lined autoclave and kept at 80 °C for 2 days. The product (Pt-MoS_2) was thoroughly washed with THF or NMP (× 2, 12,000 rpm, 10 mins), isopropanol (× 2), ethanol (× 2) and finally water (× 3) before dried in 80 °C oven. No gas bubble was observed during washing process. Ru-MoS_2, Pd-MoS_2 and Au-MoS_2 catalysts were obtained by the same procedure by using $RuCl_3·xH_2O$ (Ru 40 ~ 49%), $PdCl_2$ (99%) and $HAuCl_4·4H_2O$ (Au ~ 52%), respectively. For $RuCl_3·xH_2O$ and $PdCl_2$, anhydrous NMP was used to improve the solubility of inorganic salts.

For reference, Li_xMoS_2 powders were taken out from glove box and added to cooled ultrapure water under hydrogen evolution. Homogeneous suspension (~1 mg mL^{-1}) was sonicated for 30 min and further purified using exhaustive dialysis for 7 days to obtain exfoliated MoS_2 nanosheets. Hydrated compounds were necessary to facilitate the sluggish metal intercalation process. Feeding ratio was carefully controlled to avoid the exfoliation of MoS_2 flakes. Successful Pt loading was also observed using anhydrous K_2PtCl_4 (> 99.9%) with extended reaction time (1 week), but many large Pt aggregates were found on the surface of MoS_2.

All electrochemical measurements were performed at room temperature in a three-electrode cell using an Ag/AgCl electrode and a Pt wire as the reference and the counter electrode, or in a three-electrode H-cell using a saturated $Hg/HgSO_4$ electrode, a carbon rod and a Nafion®-117 membrane as the reference, the counter electrode and separated membrane. A glassy carbon (GC) electrode for working electrode (3 mm diameter) was polished using 3 μm, 1 μm diamond and 0.05 μm alumina slurries, followed by rinsing with ultrapure water, ethanol, acetone and ultrapure water. Finally, the GC electrode was dried under a continuous nitrogen stream. The catalyst ink was prepared by dispersing 2.0 mg catalyst in 2 mL 4:1 ethanol/water mixture with 5% Nafion® solution (20 μL) and sonicated for at least 2 hours. A quantity of 5 μL of the mixture was pipetted onto the GC electrode surface (70 μg cm^{-2} loading). Working electrode was then dried at room temperature in air for a few hours.

For HER measurements, linear sweep voltammetry (LSV) with a scan rate of 2 mV s^{-1} was recorded in 0.5 M H_2SO_4 electrolyte on a CHI 660E electrochemical workstation at room temperature. An average of at least 5 LSV curves was employed to calculate the Tafel slope. Chronoamperometry was tested at 50 mA cm^{-2} for designed period (60,000 or 120,000 s). LSV curves were recorded after operation. Electrochemical impedance spectroscopy (EIS) were taken from 4 MHz to 0.1 Hz with an amplitude of 10 mV under various overpotentials on Zahner Zennium workstation. The significance level (p-value) is < 0.05 on the average of

6 measurements. Data fittings were performed on ZView2 (Version 3.1) using a modified Randles circuit. Electrolyte was purged with N_2 for 15 min before every measurement. All potentials were calibrated with respect to reversible hydrogen electrode (RHE).

1mM fullerene-C_{60} (sublimed) was dissolved in 1:5.4 v/v acetone nitrile/toluene solution with 0.1 M tetrabutylammonium hexafluorophosphate (TBA-PF_6) as support electrolyte and 0.2 mM ferrocene (Fc) as internal standard. A 3-electrodes configuration similar to HER measurement was used here where the reference electrode was an Ag/0.01 M $AgNO_3$ electrode filled with 0.1 M TBA-PF_6 in acetone nitrile. All measurements were recorded at -15 ^oC under N_2 and Ohmic resistance was compensated 100% in all cases. *(24, 25)* CVs were recorded at 25 mV s^{-1} from 1.0 to -3.5 V (*vs.* Ag/Ag^+). DPVs were measured with 50 mV pulse, 1 ms pulse width, 300 ms period and 4 mV step E from 1.0 to -3.5 V (*vs.* Ag/Ag^+). The loading amount of 2H-MoS_2 and 1T′-Li_xMoS_2 on glassy carbon was fixed to 2 mg cm^{-2}. All potentials were referenced to the ferrocene/ferrocenium (Fc/Fc^+) couple.

Stripping experiments were performed in a three-compartment with a carbon counter and a saturated calomel electrode (SCE) reference electrode at r. t. Carbon monoxide (CO) was bubbled through the 0.5 M H_2SO_4 electrolyte with the electrode held at 0.3 V for 600 s. The solution was then purged with N_2 for an additional 900 s before a linear voltammetric scan was initiated from 0 to 1.1 V at 10 mV s^{-1}. Cu underpotential deposition (UPD) was carried out in 0.5 M H_2SO_4 and 1 mM $CuSO_4$. After cleaning and transfer into solution containing dissolved cupric ions, the electrode was polarized at 0.275 V for 300 s. CV pattern was then performed at 10 mV s^{-1} from 0.275 to 0.8 V. Charges obtained from CO stripping or Cu UPD were corrected for double layer capacity by subtracting the charge obtained for the same electrode under the same condition in N_2 without cupric. Electrochemically active surface area (ECSA) was calculated with an empirical value of 0.7 ML for saturated CO coverage, 152 μC cm^{-2} for CO monolayer oxidation as well as 420 μC cm^{-2} for Cu monolayer deposition. LSV curve was recorded prior to any measurement to ensure the HER activity.

ToF-SIMS was performed on ION-TOF $SIMS^5$ with Bi^+ primary beam (25 keV with 80x80 um^2 spot size) and Cs^+ secondary gun (2 keV, 70 nA with 230 x230 um^2 analyse area). A prolonged n-BuLi pretreatment (1 week) and Pt precursor reaction (3 days) were adopted for single crystal MoS_2 flake due to relatively slow intercalation. Pt-MoS_2 flake was then peeled off using Scotch tape method and transferred onto 300 nm SiO_2/Si substrates for mapping.

30 mg sample was first ground into fine powder using mortar and pestle in glove box, mixed with 90 mg boron nitride and pressed into a 13 mm pellet. Sample pellet was then coated with one or two drops of paraffin wax and stored under vacuum to protect from the exposure of air. XAFS measurements were performed at the 1W1B-XAFS beamline of the Beijing Synchrotron Radiation Facility (BASF). Data analysis and simulation were performed on Athena, Artemis and Hephaestus (Version 0.9.23). *(26)*

Pt-MoS_2 flake prepared from single-crystal MoS_2 was also used for GIXRD and SAXS. GIXRD was performed on a Bruker GADDS diffractometer with an area detector under Cu K_α (1.5418 Å) radiation (40 kV, 40 mA) at room temperature. The incident angle of primary beam to sample surface was moved from 0.5 to 4°, with a detection angle of 1.3 to 30°.

SAXS measurement was conducted on SAXSess mc2 (Anton Paar) from 0.08 to 5° with Cu K_α (1.5418 Å) at 40 kV and 50 mA. Pt-MoS_2 and single-crystal MoS_2 flakes were directly placed on test holder for both GIXRD and SAXS measurements. *(27)*

Supplementary References

1. Voiry, D. *et al.* Conducting MoS_2 nanosheets as catalysts for hydrogen evolution reaction. *Nano Lett.* **13**, 6222-6227 (2013).

2. Chung, D. Y. *et al.* Edge-exposed MoS_2 nano-assembled structures as efficient electrocatalysts for hydrogen evolution reaction. *Nanoscale* **6**, 2131-2136 (2014).

3. Lukowski, M. A. *et al.* Enhanced hydrogen evolution catalysis from chemically exfoliated metallic MoS_2 nanosheets. *J. Am. Chem. Soc.* **135**, 10274-10277 (2013).

4. Seo, B. *et al.* Monolayer-precision synthesis of molybdenum sulfide nanoparticles and their nanoscale size effects in the hydrogen evolution reaction. *ACS Nano* **9**, 3728-3739 (2015).

5. Wang, H. *et al.* Electrochemical tuning of vertically aligned MoS_2 nanofilms and its application in improving hydrogen evolution reaction. *Proc. Natl. Acad. Sci.* USA **110**, 19701-19706 (2013).

6. Kibsgaard, J., Jaramillo, T. F. & Besenbacher, F. Building an appropriate active-site motif into a hydrogen-evolution catalyst with thiomolybdate $[Mo_3S_{13}]^{2-}$ clusters. *Nat. Chem.* **6**, 248-253 (2014).

7. Kibsgaard, J., Chen, Z., Reinecke, B. N. & Jaramillo, T. F. Engineering the surface structure of MoS_2 to preferentially expose active edge sites for electrocatalysis. *Nat. Mater.* **11**, 963-969 (2012).

8. Chen, W. F. *et al.* Highly active and durable nanostructured molybdenum carbide electrocatalysts for hydrogen production. *Energy Environ. Sci.* **6**, 943-951 (2013).

9. Liang, H. W. *et al.* Molecular metal-N_x centres in porous carbon for electrocatalytic hydrogen evolution. *Nat. Commun.* **6**, 7992 (2015).

10. Jaramillo, T. F. *et al.* Identification of active edge sites for electrochemical H_2 evolution from MoS_2 nanocatalysts. *Scienc*e **317**, 100-102 (2007).

11. Wu, H. B., Xia, B. Y., Yu, L., Yu, X. Y. & Lou, X. W. D. Porous molybdenum carbide nano-octahedrons synthesized via confined carburization in metal-organic frameworks for efficient hydrogen production. *Nat. Commun.* **6**, 6512 (2015).

12. Bai, S. *et al.* Surface polarization matters: enhancing the hydrogen - evolution reaction by shrinking Pt shells in Pt-Pd-graphene stack structures. *Angew. Chem. Int. Edit.* **53**, 12120-12124 (2014).

13. Shi, Y. *et al.* Hot electron of Au nanorods activates the electrocatalysis of hydrogen evolution on MoS_2 nanosheets. *J. Am. Chem. Soc.* **137**, 7365-7370 (2015).

14. Zeng, Z., Tan, C., Huang, X., Bao, S. & Zhang, H. Growth of noble metal nanoparticles on single-layer TiS_2 and TaS_2 nanosheets for hydrogen evolution reaction. *Energy Environ. Sci.* **7**, 797-803 (2014).

15. Huang, X. *et al.* Solution-phase epitaxial growth of noble metal nanostructures on

dispersible single-layer molybdenum disulfide nanosheets. *Nat. Commun.* **4**, 1444 (2013).

16. Deng, J. *et al.* Triggering the electrocatalytic hydrogen evolution activity of the inert two-dimensional MoS_2 surface via single-atom metal doping. *Energy Environ. Sci.* **8**, 1594-1601 (2015).

17. Geng, F. *et al.* Unusually stable ~ 100-fold reversible and instantaneous swelling of inorganic layered materials. *Nat. Commun.* **4**, 1632 (2013).

18. Yu, X. & Ye, S. Recent advances in activity and durability enhancement of Pt/C catalytic cathode in PEMFC: Part I. Physico-chemical and electronic interaction between Pt and carbon support, and activity enhancement of Pt/C catalyst. *J. Power Sources* **172**, 133-144 (2007).

19. Yu, X. & Ye, S. Recent advances in activity and durability enhancement of Pt/C catalytic cathode in PEMFC: Part II: Degradation mechanism and durability enhancement of carbon supported platinum catalyst. *J. Power Sources* **172**, 145-154 (2007).

20. Lukowski, M. A. *et al.* Highly active hydrogen evolution catalysis from metallic WS_2 nanosheets. *Energy Environ. Sci.* **7**, 2608-2613 (2014).

21. Jiang, Z. Z., Wang, Z. B., Chu, Y. Y., Gua, D. M. & Yin, G. P. Carbon riveted microcapsule Pt/MWCNTs-TiO_2 catalyst prepared by in situ carbonized glucose with ultrahigh stability for proton exchange membrane fuel cell. *Energy Environ. Sci.* **4**, 2558-2566 (2011).

22. Herrero, E., Buller, L. J. & Abruna, H. D. Underpotential deposition at single crystal surfaces of Au, Pt, Ag and other materials. *Chem. Rev.* **101**, 1897-1930 (2001).

23. Scortichini, C. L. & Reilley, C. N. Surface characterization of Pt electrodes using underpotential deposition of H and Cu: Part I. Pt(100). *J. Electroanal. Chem.* **139**, 233-245 (1982).

24. Xie, Q., Ptrez-Cordero, E. & Echegoyen, L. Electrochemical detection of C_{60}^{6-} and C_{70}^{6-}: Enhanced stability of fullerides in solution. *J. Am. Chem. Soc.* **114**, 3978-3980 (1992).

25. Reed, C. A. & Bolskar, R. D. Discrete fulleride anions and fullerenium cations. *Chem. Rev.* **100**, 1075-1120 (2000).

26. Ravel, B. & Newville, M. ATHENA, ARTEMIS, HEPHAESTUS: data analysis for X-ray absorption spectroscopy using IFEFFIT. *J. Synchrotron Radiat.* **12**, 537-541 (2005).

27. Cook J. B. *et al.* Pseudocapacitive charge storage in thick composite MoS_2 nanocrystal-based electrodes. *Adv. Energy Mater.* **6**, 1501937 (2016).
